# Sleep disorders in people with type 2 diabetes and associated health outcomes: a review of the literature

**DOI:** 10.1007/s00125-021-05541-0

**Published:** 2021-08-16

**Authors:** Samantha B. J. Schipper, Maaike M. Van Veen, Petra J. M. Elders, Annemieke van Straten, Ysbrand D. Van Der Werf, Kristen L. Knutson, Femke Rutters

**Affiliations:** 1 Department of Epidemiology and Data Science, Amsterdam UMC, location VUmc, Amsterdam, the Netherlands; 2 Amsterdam Public Health Research Institute, Amsterdam, the Netherlands; 3 Centre of Expertise on Sleep and Psychiatry, GGZ Drenthe Mental Health Institute, Assen, the Netherlands; 4 Centre of Expertise on Sleep and Psychiatry, GGZ Drenthe Mental Health Institute, Assen, the Netherlands; 5 Department of General Practice, Amsterdam UMC, location VUmc, Amsterdam, the Netherlands; 6 Faculty of Behavioural and Movement Sciences, Vrije Universiteit, Amsterdam, the Netherlands; 7 Department of Anatomy & Neurosciences, Amsterdam UMC, Amsterdam, the Netherlands; 8 Amsterdam Neuroscience, Amsterdam, the Netherlands; 9 Department of Medicine, University of Chicago, Chicago, IL USA

**Keywords:** Health outcomes, Prevalence, Review, Sleep disorders, Type 2 diabetes

## Abstract

Sleep disorders are linked to development of type 2 diabetes and increase the risk of developing diabetes complications. Treating sleep disorders might therefore play an important role in the prevention of diabetes progression. However, the detection and treatment of sleep disorders are not part of standardised care for people with type 2 diabetes. To highlight the importance of sleep disorders in people with type 2 diabetes, we provide a review of the literature on the prevalence of sleep disorders in type 2 diabetes and the association between sleep disorders and health outcomes, such as glycaemic control, microvascular and macrovascular complications, depression, mortality and quality of life. Additionally, we examine the extent to which treating sleep disorders in people with type 2 diabetes improves these health outcomes. We performed a literature search in PubMed from inception until January 2021, using search terms for sleep disorders, type 2 diabetes, prevalence, treatment and health outcomes. Both observational and experimental studies were included in the review. We found that insomnia (39% [95% CI 34, 44]), obstructive sleep apnoea (55–86%) and restless legs syndrome (8–45%) were more prevalent in people with type 2 diabetes, compared with the general population. No studies reported prevalence rates for circadian rhythm sleep–wake disorders, central disorders of hypersomnolence or parasomnias. Additionally, several cross-sectional and prospective studies showed that sleep disorders negatively affect health outcomes in at least one diabetes domain, especially glycaemic control. For example, insomnia is associated with increased HbA_1c_ levels (2.51 mmol/mol [95% CI 1.1, 4.4]; 0.23% [95% CI 0.1, 0.4]). Finally, randomised controlled trials that investigate the effect of treating sleep disorders in people with type 2 diabetes are scarce, based on a small number of participants and sometimes inconclusive. Conventional therapies such as weight loss, sleep education and cognitive behavioural therapy seem to be effective in improving sleep and health outcomes in people with type 2 diabetes. We conclude that sleep disorders are highly prevalent in people with type 2 diabetes, negatively affecting health outcomes. Since treatment of the sleep disorder could prevent diabetes progression, efforts should be made to diagnose and treat sleep disorders in type 2 diabetes in order to ultimately improve health and therefore quality of life.

**Graphical abstract:**

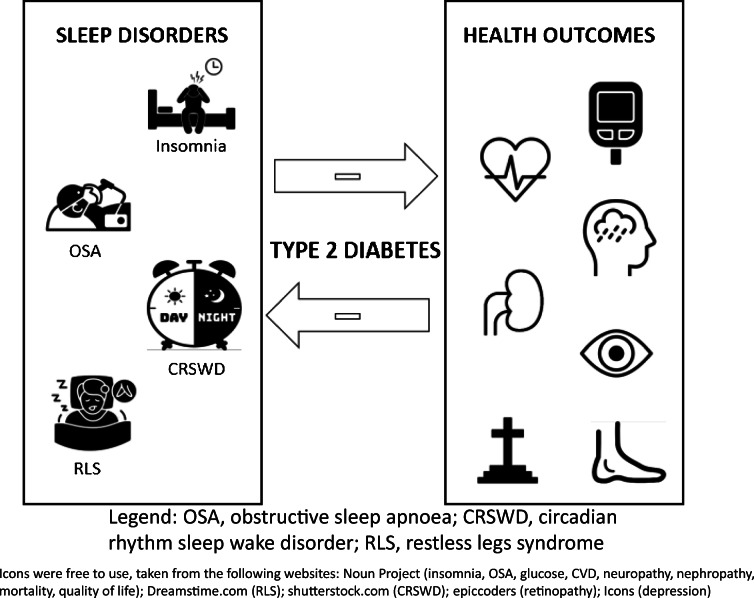

**Supplementary Information:**

The online version contains peer-reviewed but unedited supplementary material including a slideset of the figures for download, available at 10.1007/s00125-021-05541-0.

## Introduction

Diabetes is a severe public health problem, negatively affecting a person’s quality of life (QoL) and health, through increasing the risk of microvascular and macrovascular complications, depression and mortality. An important but less-known risk factor for the development of type 2 diabetes is having a sleep disorder. Sleep disorders negatively affect sleep quality and duration, causing detrimental effects on glucose metabolism and weight regulation [1]. For example, a sleep duration of <5 h and poor sleep quality are associated with developing type 2 diabetes (RR 1.48 [95% CI 1.25, 1.76] and RR 1.40 [95% CI 1.21, 1.63], respectively) [1], and insomnia and obstructive sleep apnoea (OSA) have been associated with developing type 2 diabetes (OR 1.07 [95% CI 1.02, 1.11] and OR 2.02 [95% CI 1.57, 2.61], respectively) [1, 2]. Managing and treating sleep disorders could therefore play an important role in the prevention of type 2 diabetes.

According to the of the International Classification of Sleep Disorders, third edition (ICSD-3) [3], sleep disorders can be divided into six main groups: insomnia; sleep-related breathing disorders; central disorders of hypersomnolence; circadian rhythm sleep–wake disorders (CRSWDs); parasomnias; and sleep-related movement disorders (see detailed descriptions in Table 1). Sleep disorders typically cause disturbances in the quality, amount and timing of sleep, resulting in impaired daytime functioning and distress. Several sleep disorders are highly prevalent in the general population (Table 1). Considering the association between sleep disorders and development of type 2 diabetes, the prevalence of sleep disorders in people with type 2 diabetes is probably higher compared with the general population.

**Table 1:** Definitions based on ICSD-3 and prevalence of sleep disorders in the general population

Sleep problem	Definition of sleep disorder based on ICSD-3	Prevalence in general population (%)^a^
Insomnia	Disorder characterised by a dissatisfaction in quality or quantity of sleep resulting in significant daytime distress. Insomnia is associated with problems initiating or maintaining sleep, frequent awakenings and the inability to return back to sleep. These complaints occur despite adequate opportunity and circumstances to sleep.	10
Sleep-related breathing disorders	Group of disorders characterised by symptoms such as snoring, fatigue, insomnia or subjective respiratory disturbances, or associated medical or psychiatric disorders in combination with ≥5 predominantly obstructive respiratory events per h of sleep, or ≥15 obstructive respiratory event per h (even in absence of symptoms). This diagnosis can be further subdivided into OSA disorders, central sleep apnoea syndromes, sleep-related hypoventilation disorders and idiopathic central alveolar hypoventilation.	3–7
Central disorders of hypersomnolence	Group of disorders characterised by subjective excessive daytime sleepiness that cannot be explained as a result of another sleep–wake disorder, resulting in daily occurrences of an insuppressible need to sleep or daytime lapses into sleep. This disorder group includes narcolepsy, idiopathic hypersomnia, insufficient sleep syndrome, and hypersomnias due to medical disorders, medication or substance and psychiatric disorder	0.02–0.18
CRSWDs	The disorders belonging to this group include delayed and advanced sleep–wake phase disorder, irregular sleep–wake rhythm disorder, non-24 h sleep–wake rhythm disorder, shift-work disorder, jet-lag disorder and circadian sleep–wake disorders not otherwise specified. The disorders are characterised by a chronic or recurrent pattern of sleep-disruption primarily caused by a change in the endogenous circadian timing system or misalignments between the endogenous circadian rhythm and the socially desired rhythm, resulting in insomnia or excessive sleepiness. It is associated with distress or functional impairment over a period of at least 3 months (except for jet-lag disorder).	7–16
Parasomnias	Parasomnias can be divided into NREM-related parasomnias, REM-related parasomnias and other parasomnias. NREM-related disorders include recurrent episodes of incomplete awakening, with abnormal responsiveness, limited or no memory or dream report, and at least partial amnesia for the episode. REM-related parasomnias occur as a consequence of state dissociation between REM sleep and being awake.	3–17
Sleep-related movement disorders	Group of disorders characterised by simple, often repeated movements during sleep. Diagnoses include RLS, PLMD, REM sleep behaviour disorder and others	5–10

^a^Data from [79]

ICSD-3, International Classification of Sleep Disorders, third edition; NREM, non-rapid eye movement

Sleep disorders may also result in faster diabetes progression and thus play an important role in diabetes management. However, detection and treatment of sleep disorders are not part of standardised care for people with type 2 diabetes. To start addressing the gap between current knowledge and clinical care, we provide a review of the literature on the prevalence of sleep disorders in people with type 2 diabetes and the association with the following health outcomes: glycaemic control, microvascular and macrovascular complications, depression, mortality and QoL. We also explore the extent to which treating sleep disorders in people with type 2 diabetes improves the above-mentioned health outcomes. With this review, we aim to highlight the importance of targeted diagnosis and treatment of sleep disorders in people with type 2 diabetes.

## Methods

A literature search was performed in PubMed from inception until January 2021, using MeSH and tiab search terms indicating sleep disorders (e.g. ‘sleep disorders’, ‘sleep wake disorders’, ‘sleep deprivation’, ‘circadian’, ‘sleep arousal’, ‘insomnia’, ‘obstructive sleep apnea’ and ‘restless legs syndrome’) and type 2 diabetes mellitus (e.g. ‘diabetes mellitus 2’ and ‘non-insulin dependent’). Additionally, search terms for prevalence, treatment and health outcomes were used (e.g. ‘morbidity’, ‘prevalence’, ‘depressive disorder’, ‘sleep drug’, ‘sleep medication’ and ‘health status’). See Electronic supplementary material Table 1 for the full list of search terms. All relevant English or Dutch language original and review studies were read by two authors (SS and FR) and summarised. Observational (cross-sectional and longitudinal) and experimental studies were included. No quality assessment or risk of bias assessment were made.

## Prevalence and health outcomes of sleep disorders in type 2 diabetes

**Fig. 1:**
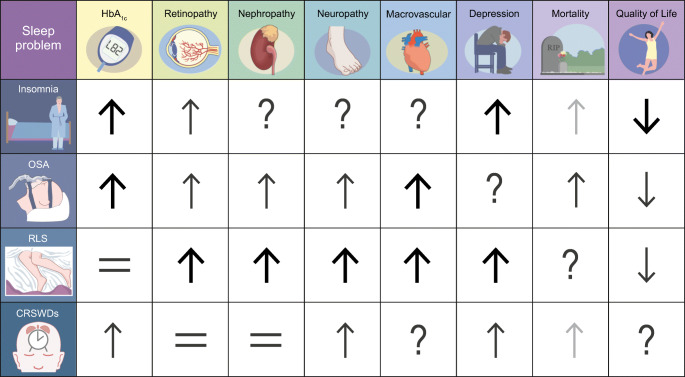
Summary of the literature to date on association between sleep disorders, health outcomes and QoL in people with type 2 diabetes. ↑, increased risk or higher levels; ↓, decreased risk or lower levels; =, no change in risk or levels; ?, no data available. Bold black arrows, strong evidence based on large study sample or multiple studies; non-bold black arrows, medium strength evidence; grey arrows, evidence based on small sample or subgroup. This figure is available as part of a downloadable slideset

In this section, we discuss the evidence on prevalence and associations of sleep disorders with health outcomes (see also Fig. 1). No or limited literature was found for hypersomnolence, parasomnias and several movement disorders. A review by Mohammadi et al. identified several papers that elaborate on mechanisms by which narcolepsy, a very specific disorder, increases the risk of development of type 2 diabetes [4]. However, no papers on prevalence of hypersomnolence and associated health outcomes in people with type 2 diabetes were identified. Only case reports were found for parasomnias (i.e. abnormal nocturnal behaviour [5] and rapid eye movement (REM) sleep behaviour disorder [6]), which will not be discussed here. For movement disorders other than restless legs syndrome (RLS) and periodic limbic movement disorder (PLMD), some were identified as a risk factor for the development of type 2 diabetes (i.e. bruxism [7]). Further research is needed to investigate the prevalence of the aforementioned sleep disorders and associated health outcomes in type 2 diabetes. Finally, data on the prevalence of having multiple sleep disorders simultaneously in those with diabetes is lacking. This is important as OSA and insomnia frequently co-occur in the general population and this co-occurrence is associated with increased comorbidities, including diabetes [8].

### Insomnia

Insomnia is characterised by difficulty initiating and maintaining sleep or by waking up earlier than desired despite adequate opportunity to sleep. A meta-analysis of 71 studies from our group [9] showed that the prevalence of insomnia and insomnia symptoms in people with type 2 diabetes is 39% (95% CI 34, 44), which is four times higher than in the general population. The prevalence was even higher with increasing age (44%) or with comorbidities present (60%). However, these numbers should be interpreted cautiously, due to the high heterogeneity of the studies included.

The meta-analysed data [9] also revealed associations between insomnia and poorer health outcomes, such as poorer control of HbA_1c_ (2.51 mmol/mol [95% CI 1.1, 4.4]; 0.23% [95% CI 0.1, 0.4]) and fasting glucose (0.4 mmol/l [95% CI 0.2, 0.7), in people with type 2 diabetes and insomnia (symptoms), compared with those with type 2 diabetes only. Chew et al. [10] showed insomnia to be associated with diabetic retinopathy (OR 1.61 [95% CI 1.01, 2.49) in 1231 people with diabetes. No studies investigated associations between insomnia and other microvascular or macrovascular complications. Our meta-analysis [9] did, however, show an association between insomnia and the predecessor of such complications (i.e. high cholesterol levels).

Several studies have shown insomnia to be associated with depressive symptoms in type 2 diabetes (OR 1.31 [95% CI 1.16, 1.47]) [11]. Furthermore, in people with diabetes or hypertension, insomnia is associated with an increased mortality rate (OR 7.17 [95% CI 1.41, 36.62]) [12]. Finally, in addition to poorer health outcomes, and perhaps most important for patients, insomnia negatively affects QoL, affecting all domains of QoL questionnaires [13, 14], compared with those with type 2 diabetes without insomnia.

### OSA

OSA, a sleep-related breathing disorder, is characterised by complaints such as non-restorative sleep, sleepiness or snoring, accompanied by obstructive respiratory events. A recent review of 12 studies, by Reutrakul and Mokhlesi [15], showed that OSA is more prevalent in people with type 2 diabetes, with the overall OSA prevalence ranging from 55% to 86%. More severe complaints and higher incidence were reported in men [15]. The prevalence of OSA is 86% in obese populations with type 2 diabetes. The shared association with obesity makes discerning an independent link between OSA and diabetes challenging. In their review, Kent et al. [16] showed that intermittent hypoxia and sleep deprivation/fragmentation play a synergistic role in glucose dysfunction and obesity. OSA is a strong predictor of diabetes, with a 49% increase in diabetes risk after adjustment for covariates, including BMI [1]. Moreover, the combined occurrence of OSA and insomnia is associated with higher prevalence of cardiometabolic morbidity, including diabetes, irrespective of BMI [8].

Although highly prevalent, OSA still remains undiagnosed in most people with type 2 diabetes managed in primary care, with only 18% being detected [15]. When diagnosed, OSA is associated with poorer glycaemic control, with 11 mmol/mol (1%) difference in HbA_1c_ levels between those with type 2 diabetes in lowest vs highest OSA severity quartiles [17]. People with type 2 diabetes and OSA are also more likely to develop microvascular complications, with OSA explaining 19% of the variance for retinopathy measures (*r* = 0.2; *p* = 0.04) [18] and being associated with an increased risk of diabetic nephropathy (OR 2.64 [95% CI 1.13, 6.16]) [19] as well as diabetic neuropathy (OR 3.97 [95% CI 1.80, 8.74]) [20]. Additionally, people with type 2 diabetes and OSA are more likely to develop coronary artery disease (HR 2.2 [95% CI 1.2, 3.9]) and heart failure (HR 3.5 [95% CI 1.4, 9.0]) [21]. No studies have reported on the association between OSA and depression in type 2 diabetes. A prospective population-based study did show that people with type 2 diabetes and OSA have a higher risk of cardiovascular mortality (HR 2.37 [95% CI 1.16, 4.82]), compared with people with only type 2 diabetes or only OSA [22]. Finally, in addition to poorer health outcomes, OSA affects QoL in people with type 2 diabetes; those with OSA score lower in all domains of QoL questionnaires, compared with people with type 2 diabetes only [23].

### RLS and PLMD

RLS is a sleep-related movement disorder that is characterised by the urge to move in response to uncomfortable and unpleasant sensations in the legs during periods of rest or inactivity, thus interfering with sleep. The exact pathophysiology of RLS is not known, but changes in dopaminergic neurotransmission, related to iron deficiency in specific brain areas, seem to play an important role [24]. Findings from 970 participants from several cross-sectional and case–control studies [25–32] suggest that the prevalence of RLS in people with type 2 diabetes ranges from 8% to 45%, based on the International RLS Study Group criteria. No sex differences were reported. This prevalence might be an overestimation, as until recently these study group criteria could not sufficiently differentiate RLS from peripheral neuropathy [25, 33].

With regard to health outcomes, in a population of 872 people with type 2 diabetes, RLS was associated with a higher prevalence of retinopathy (OR 1.69 [95% CI 1.15, 2.49]), neuropathy (OR 1.37 [95% CI 1.44, 3.90]) and nephropathy (OR 2.19 [95% CI 1.31, 3.68]) [34]. In the same population, a higher prevalence of macrovascular complications was observed, namely coronary heart disease (OR 1.95 [95% CI 1.32, 2.89]) and stroke (OR 2.15 [95% CI 1.27, 2.63]), compared with the prevalence in people with type 2 diabetes only. No statistically significant increase in HbA_1c_ level [26, 29, 31] was reported in people with type 2 diabetes and RLS, compared with those without RLS, but they were more likely to develop depression (OR 3.21 [95% CI 1.07, 11.23]) [35]. Studies on mortality, type 2 diabetes and RLS were not identified. In addition to poorer health outcomes, people with RLS and type 2 diabetes have significantly lower QoL (e.g. vitality score 52.3 vs 74.4; *p* < 0.001) than people with type 2 diabetes alone [30].

PLMD is repetitive cramping or jerking of the legs during sleep. It is the only movement disorder that occurs exclusively during sleep and is often linked with RLS, which occurs during wakefulness. Rizzi et al. [36] reported that PLMD prevalence was higher in people with type 2 diabetes than in age-matched healthy volunteers (85% vs 33%). PLMD was associated with a higher prevalence of daytime somnolence (50% vs 8%; *p* < 0.01) in type 2 diabetes, but no other studies on health outcomes related to PLMD were found.

Overall, these studies on RLS and PLMD show that sleep-related movement disorders are highly prevalent in type 2 diabetes and have negative health outcomes.

### CRSWDs

CRSWDs affect the timing of sleep either through a dysfunctional biological clock system or through a misalignment between endogenous and exogenous cues. The role of the disturbance of the circadian clock in type 2 diabetes development has been studied extensively in animals and sparsely in humans [37]. To our knowledge, there are no studies on the prevalence or related health outcomes of CRSWDs in people with type 2 diabetes. Shift work, however, is a strong predictor of CRSWDs. People with type 2 diabetes that perform (night)shift work are more likely to have insufficient glycaemic control when compared with people with type 2 diabetes performing day work: blood glucose levels of ≤7.2 mmol/l during the last 6 months (84.2% vs 71.7%; *p* = 0.02) [38]; and higher HbA_1c_ levels [39, 40].

In addition, these shift workers report poorer mental health based on the General Health Questionnaire (37.5% vs 14.2%) and more microvascular complications (e.g. higher frequency of diabetic neuropathy [10.5% vs 3.9%; *p* = 0.005] [39]), compared with people with type 2 diabetes working dayshifts. Studies reporting on macrovascular complications or QoL associated with shift work in people with type 2 diabetes are lacking. Even though based on only eight deaths, a Swedish study in over 18,000 nurses did find a significant association between nightshift work and risk of diabetes-related mortality (HR 12.0 [95% CI 3.17, 45.2]) [41]. Overall, research shows poorer health outcomes in participants with type 2 diabetes working shifts.

## Treating sleep disorders in type 2 diabetes

**Fig. 2:**
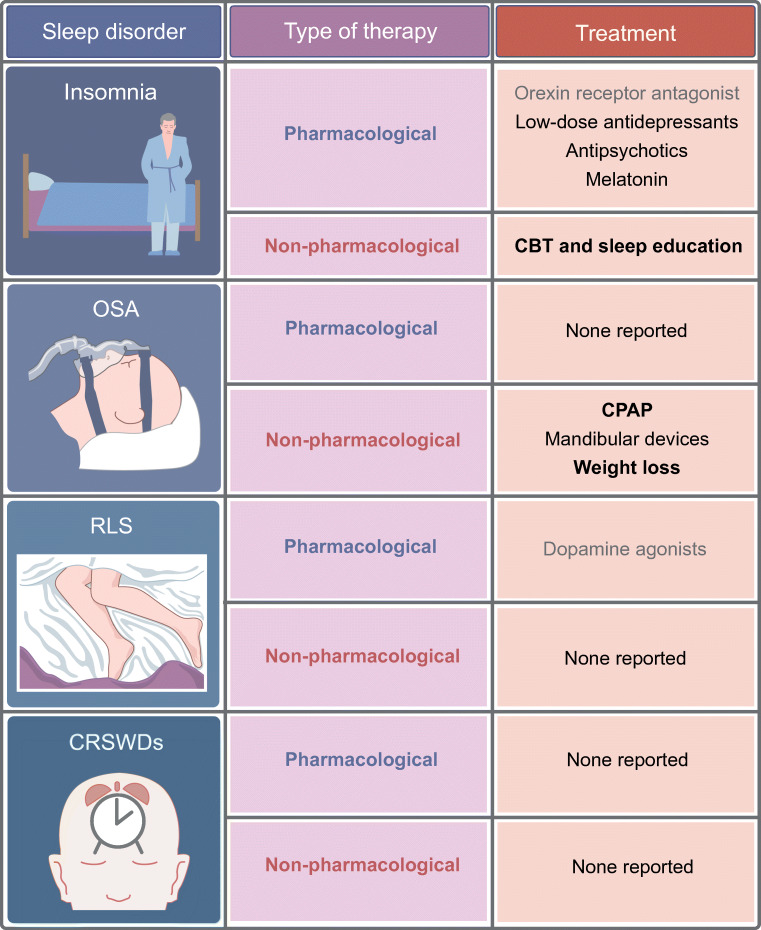
Summary of the possible pharmacological and non-pharmacological treatment options for sleep disorders in people with type 2 diabetes. Bold text, strong evidence based on large study sample or multiple studies; non-bold black text, medium strength evidence; grey text, evidence based on small study sample or subgroup. This figure is available as part of a downloadable slideset

In this section we discuss the current knowledge on treating sleep disorders in people with type 2 diabetes and the effect of treatment on health outcomes (see also Fig. 2). Sleep disorder treatments are categorised into pharmacological and non-pharmacological [42].

### Pharmacological treatment

In one study, suvorexant, a selective orexin receptor antagonist, improved sleep quality and obesity-associated variables in people with type 2 diabetes (*n* = 13) after 14 weeks [43]. In another study in 75 people with type 2 diabetes and insomnia receiving dexzopiclone or estazolam for 14 days, sleep was improved in both groups but only fasting glucose was significantly reduced in the dexzopiclone group. This suggests a direct effect of dexzopiclone on glycaemic control [44]. To date, no studies on the glycaemic effects of other hypnotics in type 2 diabetes have been published. This would be important as hypnotics are often prescribed to people with type 2 diabetes [45]. Especially considering the frequent off-label use of low-dose antidepressants or antipsychotics with known metabolic side-effects as treatment for insomnia [46], special attention is warranted regarding the health effects of such medication in people with type 2 diabetes.

Although melatonin is generally not recommended for treatment of insomnia in adults [47], two studies [48, 49] investigated the effect of a melatonin agonist or prolonged-release melatonin in people with type 2 diabetes and insomnia. The former study (*n* = 36) found that sleep quality and HbA_1c_ improved, compared with the placebo, in people with type 2 diabetes [48], whilst the latter study (*n* = 42) showed an improvement in sleep quality and stopped HbA_1c_ further increasing [49]. A meta-analysis on melatonin, also including studies in the general population, supports this finding, showing improved glycaemic control through melatonin supplementation [50]. Although this seems promising, the exact role of melatonin in type 2 diabetes is still controversial. Studies have identified a common variant of melatonin receptor 1B to be associated with impaired insulin secretion in the presence of melatonin. This suggests that melatonin could induce insulin insensitivity in the risk allele carriers and thus have adverse effects [51].

For RLS, dopamine agonists are the first-line pharmacological option [52]. In a study of the RLS population, which included people with type 2 diabetes, treatment with pramipexole reduced RLS complaint scores (−14.2 ± 0.7 vs −8.1 ± 0.7) and improved mood, compared with placebo [53]. One small Japanese study [27] examined the efficacy of pramipexole in eight people diagnosed with type 2 diabetes and RLS. A decrease in RLS scores (−13.6 points [95% CI −15.5, −11.7]) over 12 weeks and a change in HbA_1c_ levels of −3.2 mmol/mol (95% CI −4.4, −2.2) (−0.29% [95% CI −0.4, −0.2]) was observed. Other pharmacological treatments for RLS, such as other dopamine agonists, opioids, benzodiazepines, anticonvulsants and iron therapy, have not been tested in type 2 diabetes for health outcomes [54].

### Non-pharmacological treatment

#### Continuous positive airway pressure and mandibular devices

Continuous positive airway pressure (CPAP) is the gold standard for treating OSA but effects on health outcomes in people with type 2 diabetes are inconsistent. On the one hand there are studies showing a significant change in HbA_1c_ (−4.4 mmol/mol [−0.4%]; *p* = 0.024) [55], whilst, on the other hand, a meta-analysis including six randomised controlled trials in 518 people with type 2 diabetes showed that CPAP did not result in a reduction of HbA_1c_ or fasting glucose [56]. These inconsistencies might be explained by the following factors: poorer disease state, with effects being more profound in people with higher HbA_1c_ levels; differences in the definition of adherence to the CPAP intervention; or CPAP use being limited to the first half of the night when non-rapid eye movement (NREM) sleep dominates, while specifically REM sleep apnoeas in the latter half are adversely associated with glycaemic control [17]. Overall, CPAP does seem to positively affect sleep quality, blood pressure, QoL and depression [15, 57], which in turn could improve type 2 diabetes management and prevent comorbidities.

Mandibular advancement devices are another treatment option for younger people with less-severe OSA. A pilot study from India (*n* = 24) showed that mandibular devices decreased HbA_1c_ levels (−151 mmol/mol [−14.01%]; *p* = 0.013) and improved sleep quality (measured by a decrease in Epworth Sleepiness Scale of 60.7 points; *p* = 0.001) over a period of 3 months [58] in people with type 2 diabetes and mild OSA. In people with type 2 diabetes, CPAP has not been compared with mandibular advancement devices yet. However, in the general population, CPAP was more effective in reducing the number of respiratory events, whereas compliance was higher for mandibular devices [59]. Finally, surgical interventions for OSA aimed at correcting underlying anatomical abnormalities in the oropharyngeal area and the effect of hypoglossal nerve stimulation have not been researched in type 2 diabetes.

#### Weight loss

Weight loss is another important intervention for OSA. A four-centre RCT [60] found that intense lifestyle interventions were more effective than the control treatment in reducing OSA complaints (apnoea–hypopnoea index, −5.4 vs +4.2 events/h; *p* = 0.000) and HbA_1c_ levels (−7.7 mmol/mol [−0.7%] vs −2.2 mmol/mol [−0.2%]; *p* = 0.000) during 1 year. Even after 10 years, the lifestyle intervention group still showed reduced OSA complaints (apnoea–hypopnoea index, −9.9 vs −5.9 events/h; *p* = 0.11). In a meta-analysis of 136 studies (*n* = 22,094 individuals) that examined comorbid health outcomes in those who had bariatric surgery for weight loss, 87.9% (1051/1195) and 76.8% (1417/1846) had no more OSA and type 2 diabetes, respectively [61].

One study examined the effect of type 2 diabetes medication associated with weight loss on OSA in type 2 diabetes [62]. This RCT in 36 individuals investigated the effect of dapagliflozin, a sodium–glucose cotransporter 2 (SGLT2) inhibitor. No significant reduction in HbA_1c_ levels compared with the control group was observed, but significant reductions were observed in OSA severity (−10.17 events/h; *p* < 0.001), as well as systolic BP (−6.11 mmHg; *p* = 0.012) and BMI (−1.21 kg/m^2^; *p* = 0.004). These results suggest that weight loss, regardless of how the weight loss was induced, could be a successful treatment for OSA and type 2 diabetes.

#### Cognitive behavioural therapy for insomnia and sleep education

An elegant meta-analysis by Kothari et al. [44] identified six studies showing that cognitive behavioural therapy for insomnia (CBT-I) and/or sleep education improved sleep quality measured by the Pittsburgh Sleep Quality Index (−1.31 [95% CI −1.83, −0.80]) and resulted in a non-significant HbA_1c_ reduction (−3.6 mmol/mol [−0.35%]; *p* = 0.13) in those with sleep disturbances or insomnia, including both the general population and people with type 2 diabetes. Only two small pilot studies on CBT-I in people with type 2 diabetes have been conducted, reporting a reduction in both HbA_1c_ levels after 3 weeks (2.8 ± 3.06 mmol/mol [0.26 ± 0.28%]) [63] and after 7 weeks (4.5 mmol/mol [0.41%]; *p* = 0.01) [64], as well as a 4.63 (*p* = 0.002) decrease in Beck Depression Inventory measures [65] in the latter cohort. The fact that CBT-I can effectively reduce depressive symptoms has been demonstrated previously outside this specific type 2 diabetes population [66].

With regard to sleep education, two studies analysed the effects of sleep education in people with type 2 diabetes, although none with diagnosed sleep disorders. One study [67] in people with type 2 diabetes and late sleeping times showed that sleep education improved sleep quality and reduced HbA_1c_ (−1.5 ± 0.55 mmol/mol [−2.29 ± 2.20%] vs −1.11 ± 0.47 mmol/mol [−2.25 ± 2.19%]; *p* < 0.05), compared with controls. The other study [68] investigating people with type 2 diabetes and abnormal or poor sleep, reported improved sleep after sleep education but no change in glycaemic control. Overall, these studies suggest that both CBT-I and sleep education could contribute to improving sleep and health outcomes in those with type 2 diabetes and sleep disorders.

Despite the limited data available, treating sleep disorders non-pharmacologically in people with type 2 diabetes seems to have a positive effect on health outcomes. This calls for more extensive research on above-mentioned treatments as well as other promising non-pharmacological interventions in those with sleep disorders and type 2 diabetes. It is important to focus on non-pharmacological treatment because of the previously mentioned negative effects that some pharmacological options may have on weight and glycaemic control [46, 69]. One interesting option is bright light therapy (BLT), which is known for its activating and synchronising effects and is used for treatment of CRSWDs and depression. A study in 83 people with type 2 diabetes and depression, showed that BLT reduced depressive symptoms (−3.9 [95% CI −9.0, 1.2] Inventory of Depressive Symptomatology points) and improved insulin sensitivity (0.15 mg/kg × min [95% CI −0.41, 0.70] measured using hyperinsulinaemic–euglycaemic clamp), although neither change was statistically significant [70].

## Possible mechanisms, clinical implications and future research

Evidence suggests that there is a bidirectional relationship between sleep disorders and type 2 diabetes, implying a vicious circle. On the one hand, sleep disorders contribute to progression of type 2 diabetes via hypothetical mechanisms, such as decreased brain glucose utilisation, altered orexin response, overactivation of the hypothalamus–pituitary–adrenal axis [71], suboptimal self-care (i.e. lower medication adherence [72]) and impaired decision-making (i.e. unhealthy diet and sedentary behaviour) [73]. Additionally, despite the fact that no literature on this topic was found, as sleep disorders disrupt multiple metabolic processes via attenuated sleep quality, sleep quantity or disturbances of the circadian rhythm, it is conceivable that they affect the efficacy of drugs aimed at lowering HbA_1c_. On the other hand, type 2 diabetes and associated comorbidities, such as obesity, nightly hypoglycaemia, increased sympathetic activity, neuropathic pain and nocturia, may contribute to the development of sleep disorders [74]. Medication may play a role as well, with metformin, for example, causing insomnia in about 1.7% of the people starting this drug [75].

Finally, sleep architecture might differ between people with and without diabetes. EEG case–control studies show that people with type 2 diabetes indeed have lower amounts of slow wave sleep (SWS) or more micro-arousal events [76, 77], compared with matched control participants. Since these studies are cross-sectional, no cause–effect relationship can be determined and the relationship may even be bidirectional. This bidirectional relationship may explain the increased prevalence of sleep disorders in people with type 2 diabetes.

In this review, we found that insomnia, OSA and RLS are more prevalent in people with type 2 diabetes than in the general population [9, 15, 25–32]. Additionally, we showed that these three sleep disorders as well as (work-related) disturbances of the circadian rhythm negatively affect health outcomes in at least one, and often multiple, diabetes domains, especially glycaemic control. Given their high prevalence and adverse consequences, it is strongly recommended to include active assessment of possible sleep disorders in management of type 2 diabetes. Sex differences should be taken into account and special attention should be given to detecting OSA, which often remains undiagnosed in type 2 diabetes. Additionally, differentiating between RLS and conditions mimicking RLS commands attention, as this may affect treatment responsiveness [53].

In general, improving sleep in people with type 2 diabetes could in turn improve glycaemic control, thus providing an important aid in preventing type 2 diabetes progression, and ultimately improve QoL [78]. Studies on the effect of treating sleep disorders specifically in people with type 2 diabetes are limited, based on small studies, or absent. Most first-line treatments for sleep disorders seem effective in people with type 2 diabetes, comparable with the general population, but with additional positive effects on type 2 diabetes and other health outcomes. Of high clinical relevance are people with type 2 diabetes who partake in shift work, who require specific guidance in terms of meal preparation and insulin regimens in order to achieve optimal glycaemic control.

This review is the first to summarise the literature on the prevalence of sleep disorders in type 2 diabetes, its health consequences and effects of treatment of sleep disorders. We could not include information on all sleep disorders and all treatment options due to gaps in the literature, and some data presented should be interpreted cautiously because they are based on few participants or specific subgroups, as is indicated in Figs 1 and 2. A general recommendation for future studies is therefore to further explore the impact of several sleep disorders and overlap between them, including CRSWDs, central disorders of hypersomnolence and parasomnias in people with type 2 diabetes. Moreover, special research focus is warranted on the effects of both pharmacological and non-pharmacological treatment options on health outcomes in type 2 diabetes.

In conclusion, sleep disorders are highly prevalent in people with type 2 diabetes, negatively affecting health outcomes. Since treatment of the sleep disorder could prevent diabetes progression, efforts should be made to diagnose and treat sleep disorders in people with type 2 diabetes in order to ultimately improve health and therefore QoL.

## Supplementary Information

ESM 1 (PDF 184 kb)

Slideset of figures (PPTX 227 kb)

## Abbreviations

BLT: Bright light therapy
CBT-I: Cognitive behavioural therapy insomnia
CPAP: Continuous positive airway pressure
CRSWD: Circadian rhythm sleep–wake disorder
OSA: Obstructive sleep apnoea
PLMD: Periodic limbic movement disorder
QoL: Quality of life
REM: Rapid eye movement
RLS: Restless legs syndrome
