# Supplementary material for: Sleep disorders in people with type 2 diabetes and associated health outcomes: a review of the literature
Source: Diabetologia. 2021 Aug 16;64(11):2367–77. doi: 10.1007/s00125-021-05541-0 (PMC8494668; doi:10.1007/s00125-021-05541-0)
Supplement: Supplementary file 1 — (PDF 184 kb) [file 125_2021_5541_MOESM1_ESM.pdf]

Electronic supplementary material

ESM table 1 : Detailed description of search terms used in Pubmed form inception until January 2021

Search	Query	Results
#5	Search: **#1 AND #2 AND #3** Filters: **Abstract, Full text, Meta-Analysis, Randomized Controlled Trial, Review, Systematic Review, Dutch, English**	390
#4	**#1 AND #2 AND #3**	1,262
#3	Search: **"Morbidity"[MeSH] OR "morbidity"[tiab] OR "prevalence"[tiab] OR "epidemiology"[tiab] OR "glycemic control*"[tiab] OR "glucose control*"[tiab] OR "Depression"[Mesh] OR "Depressive Disorder"[Mesh:NoExp] OR "Depressive Disorder, Major"[Mesh] OR depress*[tiab] OR "cardiovascular disease*"[tiab] OR "CVD" [tiab] OR "cardiovascular diseases"[MeSH] OR "mortality"[tiab] OR "CPAP"[tiab] OR "drug therapy"[MeSH] OR "drug therap*"[tiab] OR "medication"[tiab] OR "sleep hygiene"[tiab] OR "dental guard*"[tiab] OR "mandibular advancement device*"[tiab] OR "melatonin"[MeSH] OR "melatonin*"[tiab] OR "benzo*"[tiab] OR "antidepressant*"[tiab] OR "antidepressive agents"[MeSH] OR "antidepressive agent*"[tiab] OR "sleep medication"[tiab] OR "sleep drug*"[tiab] OR "Quality of Life"[Mesh] OR "quality of life"[tiab] OR life qualit*[tiab] OR "living qualit*"[tiab] OR "quality of living"[tiab] OR "Activities of Daily Living"[Mesh] OR "activities of daily living"[tiab] OR "activity of daily living"[tiab] OR "activities of daily life"[tiab] OR "activity of daily life"[tiab] OR daily living activit*[tiab] OR daily life activit*[tiab] OR "adl"[tiab] OR "chronic limitation of activity"[tiab] OR self care*[tiab] OR "Health Status"[Mesh] OR "health status"[tiab] OR "level of health"[tiab] OR health level*[tiab] OR "qol"[tiab] OR "hrql"[tiab] OR "hrqol"[tiab] OR "Outcome Assessment, Health Care"[Mesh] OR "health outcome"[tiab]** Filters: **Abstract, Full text**	4,587,966
#2	Search: **"Diabetes Mellitus, Type 2"[Mesh] OR ((diabetes[tiab] OR diabetic*[tiab]) AND (non insulin depend*[tiab] OR noninsulin depend*[tiab] OR noninsulindepend*[tiab] OR non insulindepend*[tiab] OR maturity onset*[tiab] OR adult onset*[tiab] OR slow onset*[tiab])) OR dm2[tiab] OR niddm[tiab] OR dm 2[tiab] OR t2d*[tiab] OR dm type 2[tiab] OR type 2 diabet*[tiab] OR dm type II[tiab] OR type two diabet*[tiab] OR type II diabet*[tiab] OR dm type II[tiab]** Filters: **Abstract, Full text**	159,462
#1	Search: **"Sleep Wake Disorders"[Mesh] OR "sleep wake disorder*"[tiab] OR "sleep disorder*"[tiab] OR "dyssomnia*"[tiab] OR "parasomnia*"[tiab] OR "insomnia*"[tiab] OR "hypersomnia*"[tiab] OR "sleep deprivation"[tiab] OR "circadian disturb*"[tiab] OR "circadian rhythm"[tiab] OR "circadian misalignment"[tiab] OR "shift work*"[tiab] OR "social jetlag"[tiab] or "advance phase*"[tiab] OR "delayed phase*"[tiab] OR "sleep arousal*"[tiab] OR "jet lag syndrome*"[tiab] OR "long sleep*"[tiab] OR "short sleep*"[tiab] OR "hypersomnolence*"[tiab] OR "OSA"[tiab] OR "obstructive sleep apnea*"[tiab] OR "central apnea*"[tiab] OR "sleep related breathing disorder*"[tiab] OR "RLS"[tiab] OR "restless legs syndrome*"[tiab] OR "restless legs*"[tiab] OR "sleep related movement disorder*" [tiab] OR "sleep movement disorder*"[tiab] OR "drug induced sleep disorder*"[tiab] OR "substance induced sleep disorder*"[tiab] OR "medication induced sleep disorder*"[tiab] OR "REM sleep*"[tiab] OR "NREM sleep*"[tiab] OR "rapid eye movement sleep*"[tiab] OR "rapid eye movement sleep parasomnia*"[tiab] OR "rem sleep behavior disorder*"[tiab] OR "sleepwalk*"[tiab] OR "sleep terror*"[tiab] OR "non-rapid eye movement disorder*"[tiab] OR "night terror*"[tiab]** Filters: **Abstract, Full text**	111,206
