# Supplementary material for: Sleep disorders in people with type 2 diabetes and associated health outcomes: a review of the literature
Source: Diabetologia. 2021 Aug 16;64(11):2367–77. doi: 10.1007/s00125-021-05541-0 (PMC8494668; doi:10.1007/s00125-021-05541-0)
Supplement: Supplementary file 2 — (PPTX 227 kb) [file 125_2021_5541_MOESM2_ESM.pptx]

## Slide 1
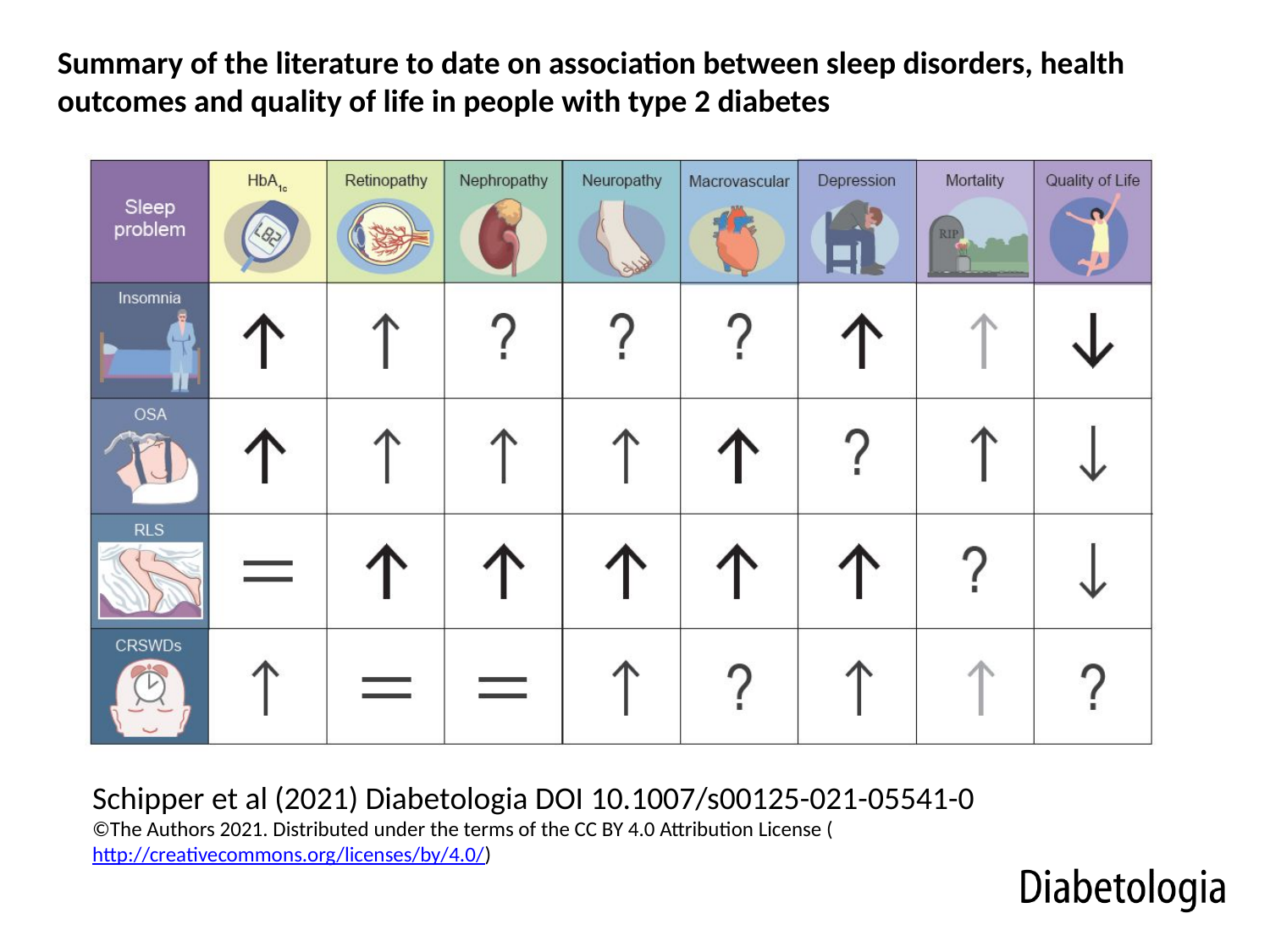

Summary of the literature to date on association between sleep disorders, health outcomes and quality of life in people with type 2 diabetes
Schipper et al (2021) Diabetologia DOI 10.1007/s00125-021-05541-0
©The Authors 2021. Distributed under the terms of the CC BY 4.0 Attribution License (http://creativecommons.org/licenses/by/4.0/)

## Slide 2
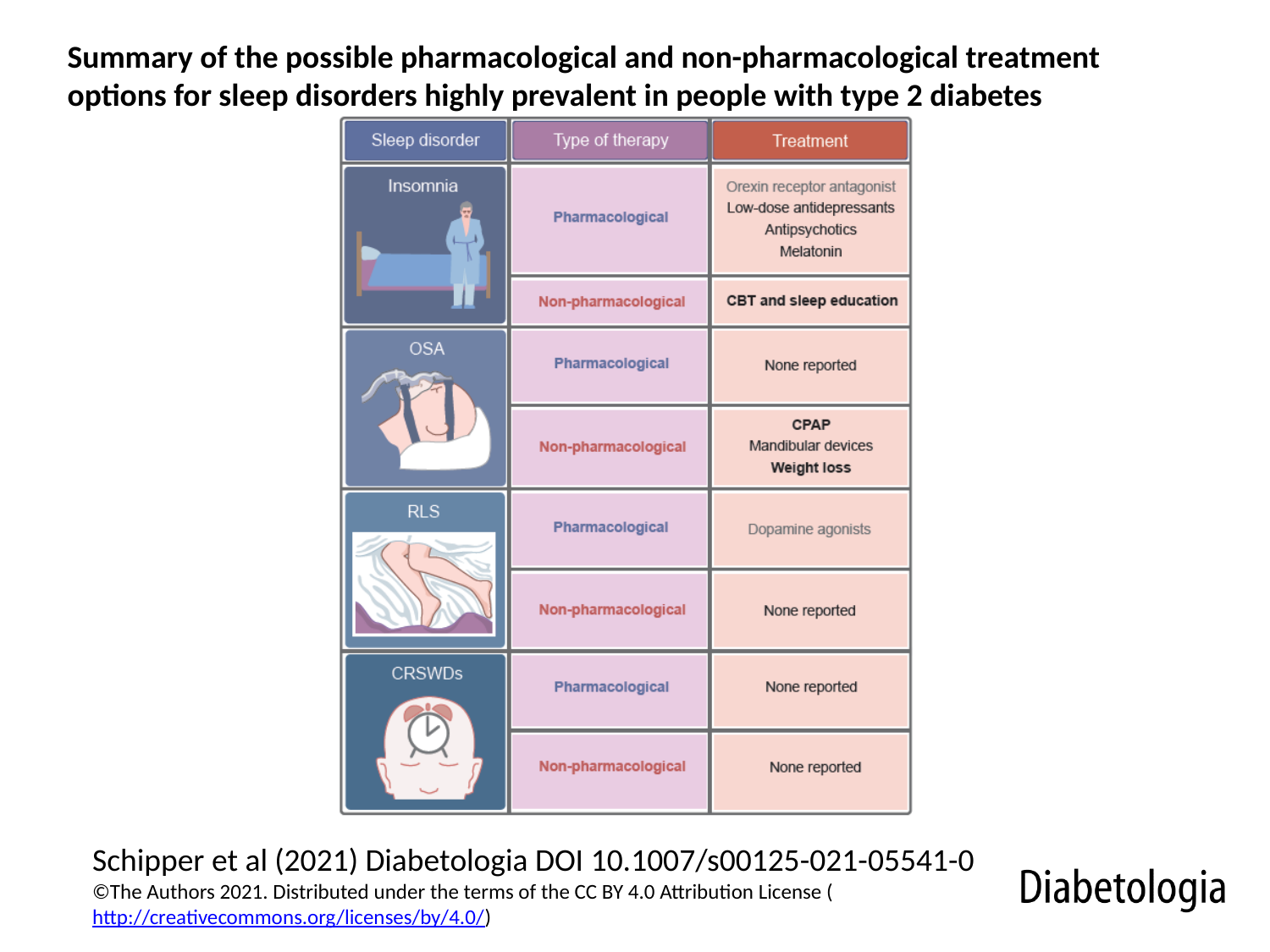

Summary of the possible pharmacological and non-pharmacological treatment options for sleep disorders highly prevalent in people with type 2 diabetes
Schipper et al (2021) Diabetologia DOI 10.1007/s00125-021-05541-0
©The Authors 2021. Distributed under the terms of the CC BY 4.0 Attribution License (http://creativecommons.org/licenses/by/4.0/)
